# Climate change impacts on thermal stress in four climatically diverse European cities

**DOI:** 10.1007/s00484-022-02361-8

**Published:** 2022-09-21

**Authors:** George Katavoutas, Dimitra Founda, Konstantinos V. Varotsos, Christos Giannakopoulos

**Affiliations:** National Observatory of Athens, Institute for Environmental Research and Sustainable Development, 15236 Athens, Greece

**Keywords:** Thermal stress, Thermal comfort, Climate change implications, Universal Thermal Climate Index, Cold/warm cities, Europe

## Abstract

The thermal conditions that prevail in cities pose a number of challenges to urban residents and policy makers related to quality of life, health and welfare as well as to sustainable urban development. However, the changes in thermal stress due to climate change are probably not uniform among cities with different background climates. In this work, a comparative analysis of observed and projected thermal stress (cold stress, heat stress, no thermal stress) across four European cities (Helsinki, Rotterdam, Vienna, and Athens), which are representative of different geographical and climatic regions of the continent, for a recent period (1975 − 2004) and two future periods (2029 − 2058, 2069 − 2098) has been conducted. Applying a rational thermal index (Universal Thermal Climate Index) and considering two models of the EURO-CORDEX experiment (RCA4-MOHC, RCA4-MPI) under two Representative Concentration Pathways (RCP4.5, RCP8.5), the projected future changes in thermal conditions are inspected. The distribution of thermal stress in the current climate varies greatly between the cities, reflecting their climatic and urban heterogeneity. In the future climate, a reduction in the frequency of cold stress is expected across all cities, ranging between − 2.9% and − 16.2%. The projected increase in the frequency of optimal thermal conditions increases with increasing latitude, while the projected increase in the frequency of heat stress (ranging from + 0.2 to + 14.6%) decreases with increasing latitudes. Asymmetrical changes in cold- and heat-related stress between cities were found to affect the annual percentage of optimal (no thermal stress) conditions in future. Although future projections are expected to partly bridge the gap between the less-privileged cities (with respect to annual frequency of optimal thermal conditions) like Helsinki and Rotterdam and the more privileged ones like Athens, the former will still lag behind on an annual basis.

## Introduction

The thermal conditions that are formed in cities in the twenty-first century pose a number of challenges to urban residents and policy makers related to quality of life, health and welfare as well as to sustainable urban development. In the years to come, urban population and urbanization levels are projected to increase (United Nations 2019) and in combination with the burden of thermal/health risk due to climate change (Ebi et al. 2018; Founda 2021; Kjellstrom et al. 2016; Matthews et al. 2017; Sheridan and Allen 2015) will further put the thermal environment of cities in the spotlight of scientific research.

Nowadays, the population residing in urban areas represents more than half of global population and almost the three-quarters of the European population (United Nations 2019). Although in general urban dwellers are sharing high standards of living (income, access to services and technology, employment opportunities), they are experiencing at the same time the deterioration of optimal thermal conditions due to the changes in the mean climate and climatic extremes in the context of climate change (Guerreiro et al. 2018; Founda et al. 2022a, b; Smid et al. 2019). In addition, the urban heat island which is enhanced during periods of excessive heat (heat waves) increases the residents’ vulnerability to thermal risk, excess mortality, and morbidity (Katavoutas and Founda 2019a; Rasilla et al. 2019; Sarangi et al. 2021; Van Hove et al. 2015; Zhao et al. 2018).

Recent studies analyzing climate observational or reanalysis data have shown evidence of degradation of optimal thermal conditions in European cities during the last decades (Antonescu et al. 2021; Di Napoli et al. 2018; Founda et al. 2019a; Katavoutas and Founda 2019b). Cities located in southern Europe have been experiencing increased frequency of heat related stress (Antonescu et al. 2021; Katavoutas and Founda 2019b), while for those in northern Europe a decrease in the frequency and persistency of cold stress is apparent (Antonescu et al. 2021; Founda et al. 2019a). Analyzing projected air temperature data from 571 European cities, Guerreiro et al. (2018) found that heat waves, namely prolonged periods of excessive heat, are expected to be more frequent and intense in the future for all cities, with southern cities experiencing the highest increase in the frequency of heat wave days and the central European cities experiencing the higher maximum temperature increases during heat waves. However, not only changes in the frequency and intensity of hot extremes have been reported but also changes in their seasonality as well as in the lengths and timing of thermal seasons, which are also of vital importance. According to the study of Ruosteenoja et al. (2020), the length of thermal summer (daily mean air temperature higher than 10 °C) in northern Europe is expected to increase by nearly 30 days on average in the future (2040–2069), while the thermal winter (daily mean air temperature lower than 0 °C) is foreseen to shorten by up to 60 days. In the eastern Mediterranean, an elongation of hot extremes’ season by more than two months is expected until the end of the twenty-first century (Founda et al. 2019b). Employing a bioclimatic index like Universal Thermal Climate Index (UTCI), Di Napoli et al. (2018) found an increase in heat stress up to 1 °C (according to the index scale) in Europe over the last decades, along with increased mortality under moderate and strong heat stress conditions.

Changes on thermal conditions due to climate change affect the human thermal stress levels; however, these changes are not uniform and the magnitude of their impacts may differ widely, especially in regions with different climatic features (Guerreiro et al. 2018; Jendritzky and Tinz 2009; Knutson and Ploshay 2016; Smid et al. 2019). In addition, our better understanding of climate change impacts on thermal stress is important, as it could lead to more targeted actions that could be adopted by the policy makers in adaptation strategies, especially in the cities whose population is particularly vulnerable to thermal risk (Smid et al. 2019).

This study aims to assess and highlight any differentiation between colder and warmer cities with respect to the observed and projected changes in thermal stress levels. This in turn could impact, either directly or indirectly, a number of key sectors like health, tourism, energy, urban planning and others. To this end, the study focuses on four European cities (Helsinki, Rotterdam, Vienna and Athens), which are representative of different geographical and climatic regions of the continent, to assess the climate change impacts on cold stress, heat stress, and no thermal stress by the end of twenty-first century. Observational and projected data derived from a Regional Climate Model (RCA4), driven by two Global Climate Models (Met Office Hadley Centre model HadGEM-ES and Max Planck Institute for Meteorology model MPI-ESM-LR) in the framework of EURO-CORDEX experiment, were employed to quantify thermal stress by applying an advanced thermal index (Universal Thermal Climate Index). The projected changes in the annual and seasonal frequency of optimal thermal conditions (no thermal stress) across cities have also been assessed and discussed. Apart from the effect on the quality of life and wellbeing of the residents, changes in the frequency of optimal thermal conditions could also lead to a possible redistribution of the tourist flows between warmer and colder cities.

## Materials and methods

### Study areas

**Fig. 1:**
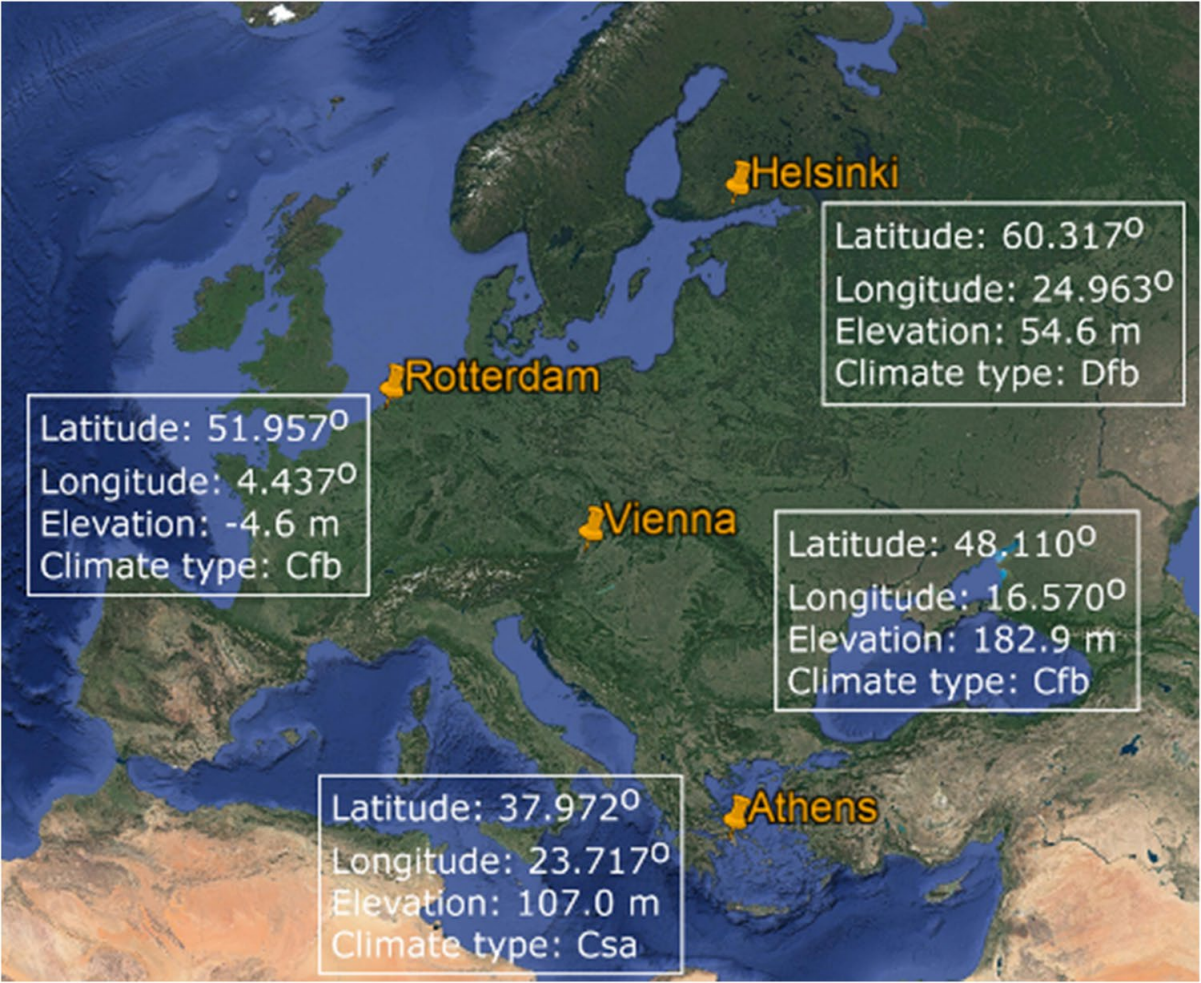
Map presenting the selected cities along with coordinates, elevation, and climate type following the Köppen-Geiger climate classification (Map was produced in Google Earth Pro; Map data SIO, NOAA, US Navy, NGA, GEBCO; Image Landsat)

Four European cities were selected in this study, representing different geographical and climatic regions of the continent (Fig. 1). The selected cities are located in Northern (Helsinki), Western (Rotterdam), Central (Vienna), and Southern Europe (Athens), experiencing different climate types. According to the updated Köppen–Geiger climate classification (Kottek et al. 2006; Peel et al. 2007), Helsinki has a boreal climate, fully humid with warm summer (Dfb). The climate in Rotterdam and Vienna is classified as warm temperate, fully humid with warm summer (Cfb). Although both cities are sharing the same type of climate, Rotterdam is located near to the coast while the continental city of Vienna is situated on the foothills of the Alps. On the contrary, Athens is located in the Mediterranean climate zone and has a warm temperate climate with dry and hot summer (Csa).

Besides different climate types, the considered cities demonstrate different demographic characteristics such as human population, population density and built-up area, which are important societal variables in the analysis of climate change impacts. The urban population has increased over the last 50 years in all four cities and nowadays approximates 907,000 in Helsinki, 1.8 million in Vienna, 1.9 million in Rotterdam and 3.3 million in Athens according to the Urban Centre Database of the European Commission (Florczyk et al. 2019). Helsinki presents the lowest population density (2638 inhabitants/km^2^) and Athens the highest (7569 inhabitants/km^2^) among the considered cities. While Vienna and Rotterdam have similar urban population, the population density in Vienna (4736 inhabitants/km^2^) is much higher compared to Rotterdam (2909 inhabitants/km^2^). Rotterdam has the largest built-up area (419km^2^), followed by Athens (326km^2^) and Vienna (247km^2^), while Helsinki has the smallest one (117km^2^).

### Observational data

Synchronous meteorological observations for Helsinki, Rotterdam and Vienna at 3-hourly temporal resolution (00:00, 03:00, 06:00, 09:00, 12:00, 15:00, 18:00, 21:00 UTC, Universal Time Coordinated) were extracted from the database of the National Oceanic and Atmospheric Administration (NOAA) (https://www.ncei.noaa.gov/access/search/data-search/global-hourly). Meteorological observations for Athens at the same temporal step were derived from the climatic records of the National Observatory of Athens. The variables of air temperature, relative humidity, wind speed, cloud cover, and global solar radiation were used covering a period of 30 years (1975 − 2004). This period was selected on the one hand due to the quality and simultaneous completeness of the data and on the other hand to serve as the reference (control) period for the comparison between observations and model simulations. The weather stations in Helsinki, Rotterdam, and Vienna are located at the homonymous international airports of these cities, which in turn are situated at the boundaries of the built-up area, reflecting the close proximity to the urban population. The station of the National Observatory of Athens is located on a small hill at the city center.

### RCM simulations

In addition to observational data, the study further employs 3-hourly air temperature, relative humidity, wind speed, and downwelling shortwave radiation data for the closest land grid point to the stations’ location from the Regional Climate Model (RCM) RCA4 of the Swedish Meteorological and Hydrological Institute (SMHI, Strandberg et al. 2015) driven by (1) the Met Office Hadley Centre model HadGEM-ES (hereafter RCA4-MOHC) (Collins et al. 2011; Martin et al. 2011) and (2) the Max Planck Institute for Meteorology model MPI-ESM-LR (hereafter RCA4-MPI) (Popke et al. 2013). The horizontal resolution of the RCA4 model is 0.11° (~ 12 × 12 km) and the simulations were carried out in the framework of the EURO-CORDEX modeling experiment (http://www.euro-cordex.net). The boundary conditions of the RCA4 SMHI regional climate model are defined on the basis of the selected global models (MOHC, MPI), making the combined modelling systems substantially diverse so as to be considered different models. The simulated parameters of air temperature, relative humidity, and wind speed are instantaneous values at the same sub daily time step and synchronous to the observations, namely at 00:00, 03:00, 06:00, 09:00, 12:00, 15:00, 18:00, and 21:00 UTC, in the studied locations. The simulated downwelling shortwave radiation represents average value of each 3-hourly interval. In order to secure the best possible synchronization with other instantaneous meteorological variables, downwelling shortwave radiation of each pair of consecutive time intervals was also averaged. Further details on CORDEX archive design can be found at https://cordex.org/publications/report-and-document-archives. The simulated data cover three 30-year time-slices, the 1975 − 2004 reference period, and the two periods 2029 − 2058 and 2069 − 2098 to represent near and distant future, respectively. Future simulations are based on two alternative greenhouse gas concentrations trajectories (Representative Concentration Pathways, RCP), the RCP4.5 and the RCP8.5. The RCP4.5 scenario assumes stabilization of the total radiative forcing shortly after 2100 and can be considered as an intermediate mitigation scenario (Thomson et al. 2011) whereas the RCP8.5 scenario represents a climate future where no policies aiming at the reduction of greenhouse gas emissions are implemented (Riahi et al. 2007; Van Vuuren et al. 2011).

### Analysis procedures of human thermal stress

To assess the levels of thermal stress of the population in the selected cities, the Universal Thermal Climate Index (UTCI) was employed. UTCI is an energy balance stress index providing a measure of thermal environmental stress imposed on the human body (de Freitas and Grigorieva 2017). It is expressed as an equivalent temperature, and it is classified into ten categories depending on thermal stress level (Table 1). UTCI is derived from a complex multi-node model taking into account the physiology of thermoregulation and the energy balance theory (Fiala et al. 2012; Jendritzky et al. 2012). This multi-node model is also accompanied with a clothing model which adjusts the parameter of clothing insulation according to the ambient temperature for light activity conditions (Havenith et al. 2012). The UTCI threshold values for each thermal stress category in Table 1 were based on a number of detailed criteria (46 in total) concerning the strain reactions of the human body, such as the onset of sweating, the onset of shivering, or based on significant changes in the responses of the thermophysiological parameters (Bröde et al. 2012).

**Table 1:** The 10-categories scale of thermal stress according to UTCI (Bröde et al. 2012)

UTCI (°C)	Stress category
(46, + ∞)	Extreme heat stress
(38, 46]	Very strong heat stress
(32, 38]	Strong heat stress
(26, 32]	Moderate heat stress
[9, 26]	No thermal stress
[0, 9)	Slight cold stress
[− 13, 0)	Moderate cold stress
[− 27, − 13)	Strong cold stress
[− 40, − 27)	Very strong cold stress
(− ∞, − 40)	Extreme cold stress

UTCI has been designed to reflect the temporary thermal stress at a specific moment in time. All parameters related to the calculations of the UTCI are instantaneous in this study, except for the simulated parameter related to radiation. UTCI was estimated from both observations and model simulations for each city based on the 3-hourly data of air temperature, water vapor pressure, wind speed, and mean radiant temperature. The latter parameter was calculated using the RayMan model, a micro-scale model that simulates longwave and shortwave radiation fluxes in the outdoor environment (Matzarakis et al. 2007, 2010). In addition to the meteorological parameters (air temperature, water vapor pressure, wind speed, cloud cover, or downwelling shortwave radiation) associated with the mean radiant temperature calculations, specific details concerning the stations’ location (longitude, latitude, elevation) and the time (time zone, local time, day of the year) for each site were used.

For the assessment of thermal comfort and the associated thermal stress levels, a large number of indices have been proposed over time. The advantage of using the UTCI is the consideration of all energy exchange mechanisms between the human body and the thermal environment, covering the full range of hot to cold environmental conditions (Jendritzky et al. 2012). This is achieved on the basis of a detailed thermo-physiological model predicting in a more reliable way the human thermal response against other models (Psikuta et al. 2012). In addition, the linkage between UTCI and mortality in Europe reveals the index importance in an era that climate change progresses (Di Napoli et al. 2018).

In the following paragraphs when referring to cold stress or heat stress without mentioning the intensity level, that signifies that all five related categories for the case of cold stress were taken into account (UTCI < 9 °C) and that all four related categories for the case of heat stress were taken into account (UTCI > 26 °C) (Table 1). The above approach was based on the adoption of the original scale of UTCI (Bröde et al. 2012), and therefore, factors such as the physiological adaptation, acclimatization, expectations, and behavior that could possibly modify the thresholds of thermal stress categories were not taken into consideration both in the current and in the future climate.

## Results

### Thermal environmental stress based on observations

**Fig. 2:**
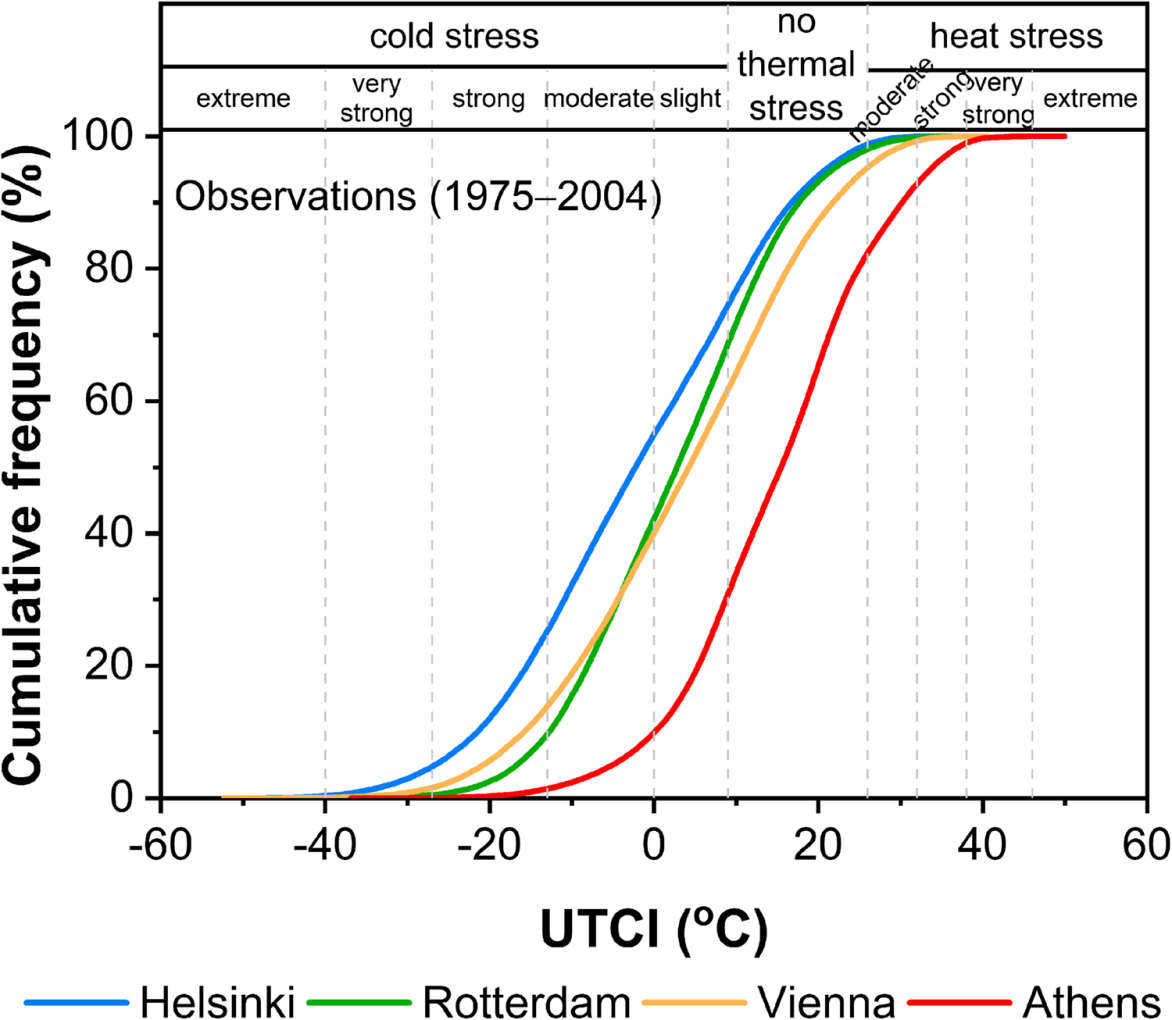
Cumulative frequency distribution of UTCI based on observational data for the reference period (1975 − 2004) in the cities of Helsinki, Rotterdam, Vienna and Athens (bin size 0.1 °C)

The cumulative frequency curves (CFCs) in Fig. 2 represent the distribution of thermal stress according to the UTCI for the 30-year reference period (1975 − 2004) in the selected cities. The distribution of thermal stress varies greatly among cities, reflecting the climatic and urban heterogeneity. For Helsinki, the center of the distribution lies within the moderate cold stress category, that is one category lower compared to Rotterdam and Vienna (slight cold stress) and two categories lower compared to Athens (no thermal stress). Approximately 75% of UTCI values in Helsinki are less than 9 °C, which is the upper threshold of cold stress. This percentage is lower in Rotterdam (69%) and Vienna (62%), and considerably lower in Athens (31%). The percentage of UTCI values that is less than − 13 °C, reflecting the most intense cold stress, is extremely small for Athens (< 1.5%), but it constitutes a significant proportion for Helsinki (~ 25%). Approximately 18% of UTCI values in Athens are higher than 26 °C, which is the lowest threshold of heat stress. The corresponding percentage is significantly lower in Vienna (5%), Rotterdam (2%), and Helsinki (1%). The Athens population is experiencing thermal conditions that impose no thermal stress on the human body at a percentage of 51%, which is almost twice as high of that observed in Helsinki (24%). The corresponding percentage for Vienna (33%) is higher than that noticed in Rotterdam (29%) and Helsinki but lower than that in Athens. It is also seen that the CFCs of Vienna and Rotterdam intersect each other (− 4 °C, 31%). Therefore, the percentage of UTCI values that are less than the intersection point is higher in Vienna than in Rotterdam, while the opposite is observed for the UTCI values that are higher of this point. Since both cities share the same type of climate, this outcome probably reflects the difference in elevation as well as the proximity of Vienna to the Alps against the proximity of Rotterdam to the coast.

### Models performance evaluation

**Fig. 3:**
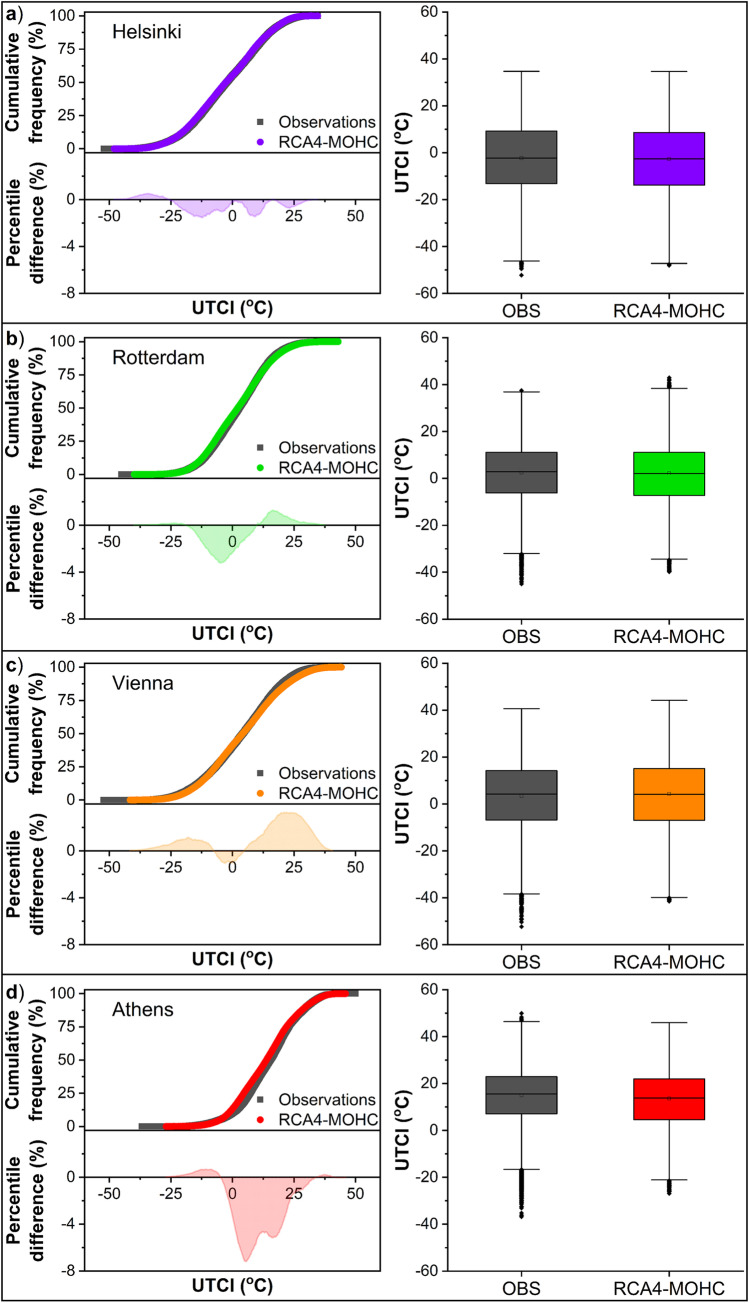
Cumulative frequency distribution of UTCI from observations and simulations according to the RCA4-MOHC model for the reference period (1975 − 2004) in the cities of **a**) Helsinki, **b**) Rotterdam, **c**) Vienna, and **d**) Athens (bin size 0.1 °C). The percentile difference between observations and simulations across the whole range of UTCI values as well as the box plots of UTCI are also illustrated

**Fig. 4:**
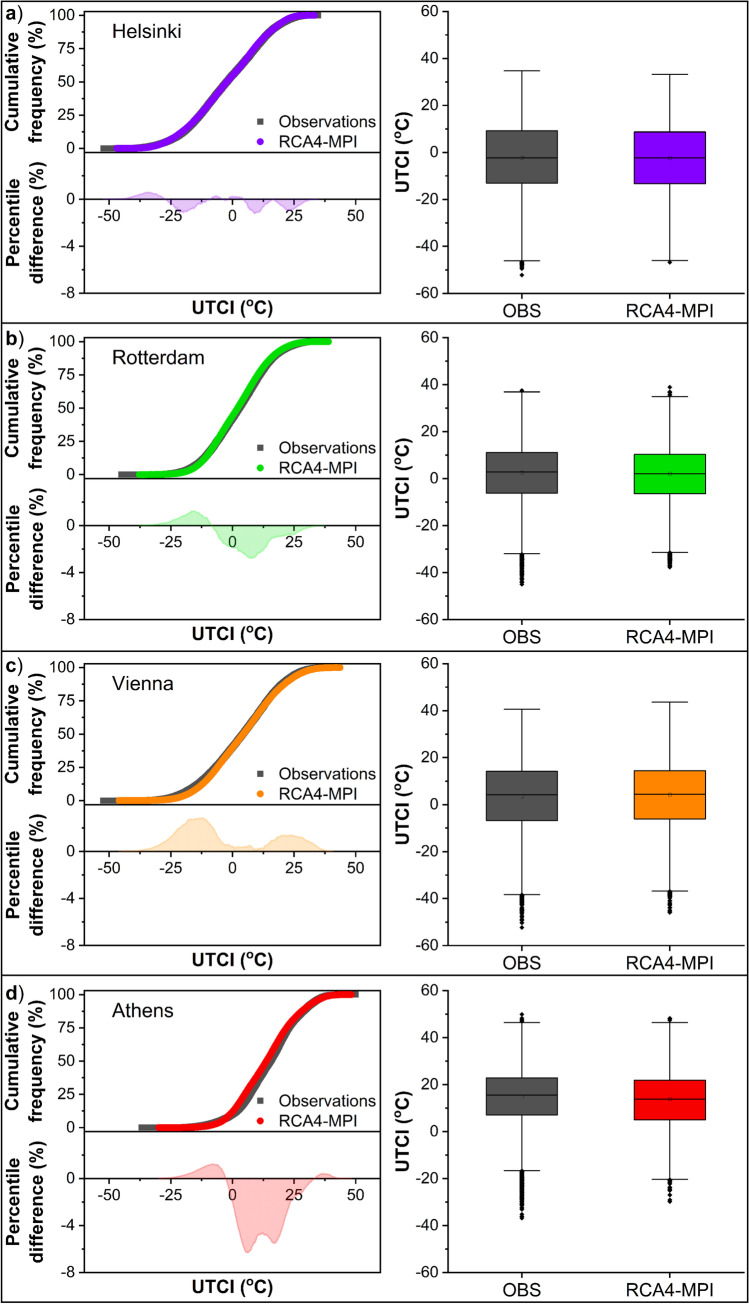
Cumulative frequency distribution of UTCI from observations and simulations according to the RCA4-MPI model for the reference period (1975 − 2004) in the cities of **a**) Helsinki, **b**) Rotterdam, **c**) Vienna, and **d**) Athens (bin size 0.1 °C). The percentile difference between observations and simulations across the whole range of UTCI values as well as the box plots of UTCI are also illustrated

In order to assess the performance of the selected models on thermal stress expressed by the UTCI, the index values derived from simulations and observations over the 30-year reference period (1975 − 2004) were compared (Figs. 3 and 4). The CFCs of UTCI based on simulations are reasonably close to the CFCs based on observations in all cities and for both models. The percentile differences (positive or negative) between observations and simulations from the RCA4-MOHC are less than 1.5 percentage points in Helsinki (Fig. 3a), 3.2 percentage points in Rotterdam and Vienna (Fig. 3b, c), and 7.0 percentage points in Athens (Fig. 3d) throughout the whole range of UTCI values. The corresponding differences for the RCA4-MPI are slightly smaller, with the lowest being observed in Helsinki (less than 1.2 percentage points) (Fig. 4a) and the highest in Athens (less than 6.3 percentage points) (Fig. 4d).

Figures 3 and 4 (right panels) present the boxplots of simulated and observed UTCI values over the reference period. An excellent agreement between the medians of simulated and observed UTCI is noticed, with absolute differences lower than 0.7 °C in all cities and models, except for Athens where the difference is 1.7 °C in both models. The interquartile ranges are also reasonably similar, as shown by the lengths of the boxes, with the difference, either positive or negative, being less than 0.3 °C in Helsinki, 1.0 °C in Rotterdam and Vienna, and 1.5 °C in Athens. Besides, the overall spreads (distances between adjacent values at the end points of whiskers) are roughly similar, though the overall range (maximum-minimum) of the UTCI values based on observations is slightly greater than that based on simulations. These results suggest a very good agreement between the observed and simulated index, and that both models capture successfully the variability of UTCI values at the sites of interest.

### Future thermal stress projections

**Fig. 5:**
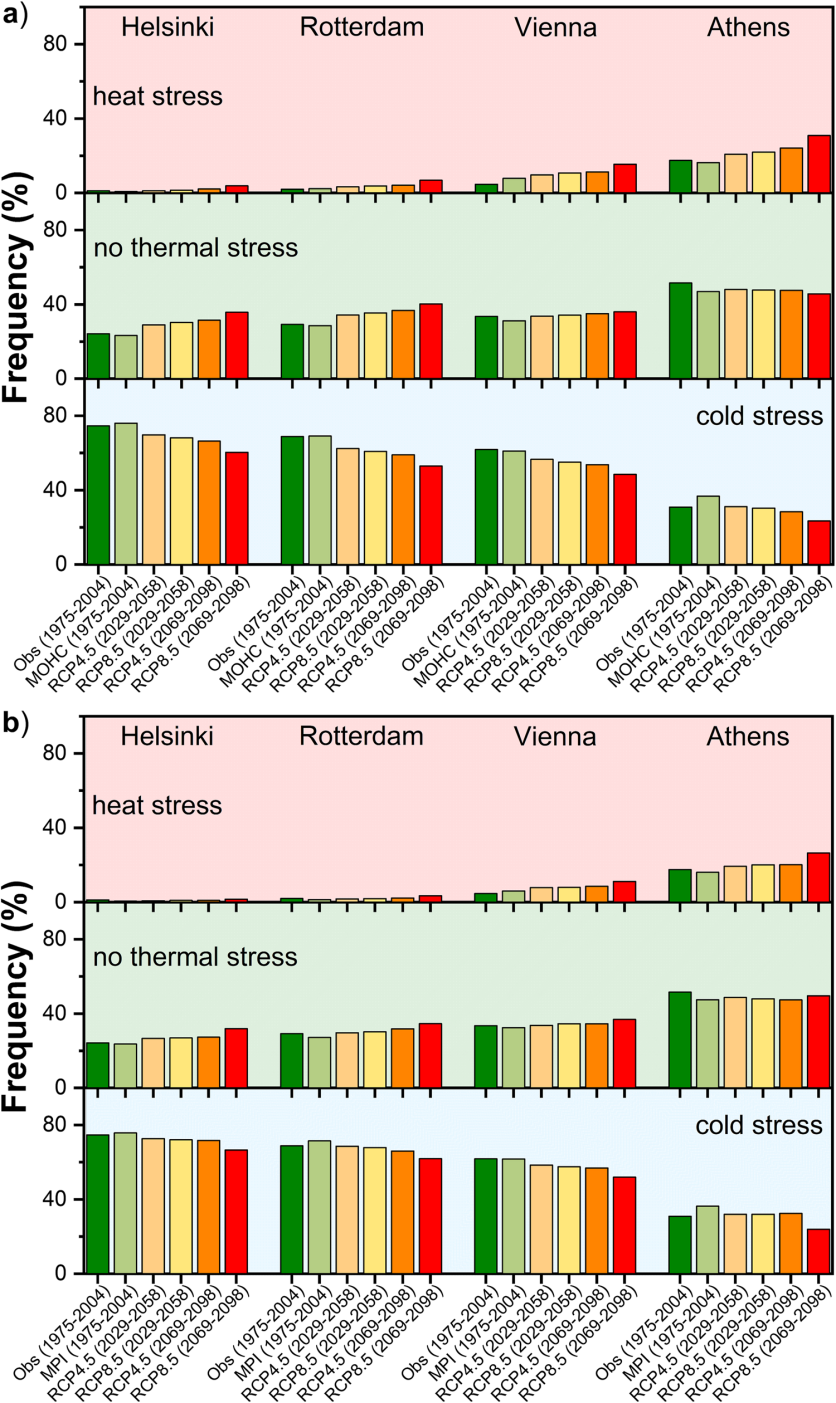
Frequency of “heat stress”, “no thermal stress”, and “cold stress” conditions based on UTCI for the reference period (1975 − 2004), the near future (2029 − 2058), and the distant future (2069 − 2098) according to the **a**) RCA4-MOHC and **b**) RCA4-MPI models for RCP4.5 and RCP8.5 in the cities of Helsinki, Rotterdam, Vienna, and Athens

Figure 5 presents the frequency (%) of heat stress (UTCI > 26 °C), no thermal stress (9 °C ≤ UTCI ≤ 26 °C) and cold stress (UTCI < 9 °C) cases in the selected European cities for the reference period (1975 − 2004), as well as for the near (2029 − 2058) and distant future (2069 − 2098) periods, under both scenarios (RCP4.5, RCP8.5) and both models RCA4-MOHC (Fig. 5a) and RCA4-MPI (Fig. 5b). In Helsinki, the frequency of cold stress is expected to decrease in the future, whereas the frequencies of no thermal stress and heat stress are projected to increase according to both models, with higher increase in no thermal stress (Fig. 5). A similar pattern is observed in Rotterdam, although quantitative differences in frequencies are noted compared to Helsinki. In Vienna, an obvious increase in the frequency of heat stress and a clear decrease in the frequency of cold stress are expected in the future periods according to both models (Fig. 5). On the other hand, the frequency of no thermal stress shows a slight increase in the future, less prominent than in Rotterdam and Helsinki, though. The frequency of heat stress in Athens is the highest among the examined cities in the reference period and is foreseen to further increase in the future according to both models (Fig. 5). At the same time, the frequency of cold stress is expected to decrease in the future, while the frequency of no thermal stress appears to stabilize in the future compared to the reference period. The latter outcome reveals a counterbalance in the frequencies between the conditions that are expected to shift from the cold stress towards the no thermal stress category and those that are foreseen to shift from the no thermal stress towards the heat stress category in the future in Athens.

**Fig. 6:**
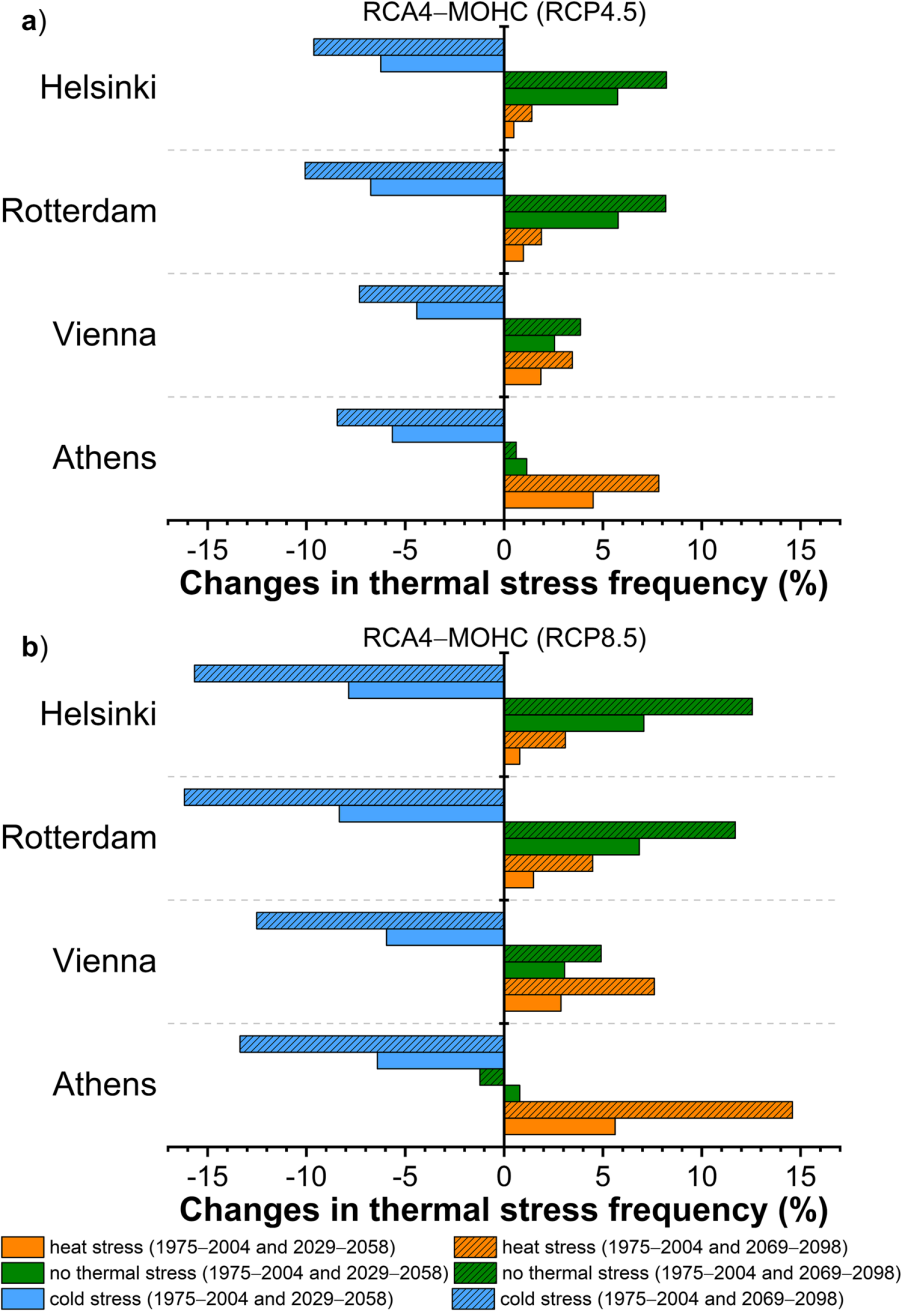
Changes in thermal stress frequency based on UTCI between the reference period (1975 − 2004) and the near future (2029 − 2058) or the distant future (2069 − 2098) according to the RCA4-MOHC model for **a**) RCP4.5 and **b**) RCP8.5 in the cities of Helsinki, Rotterdam, Vienna, and Athens

**Fig. 7:**
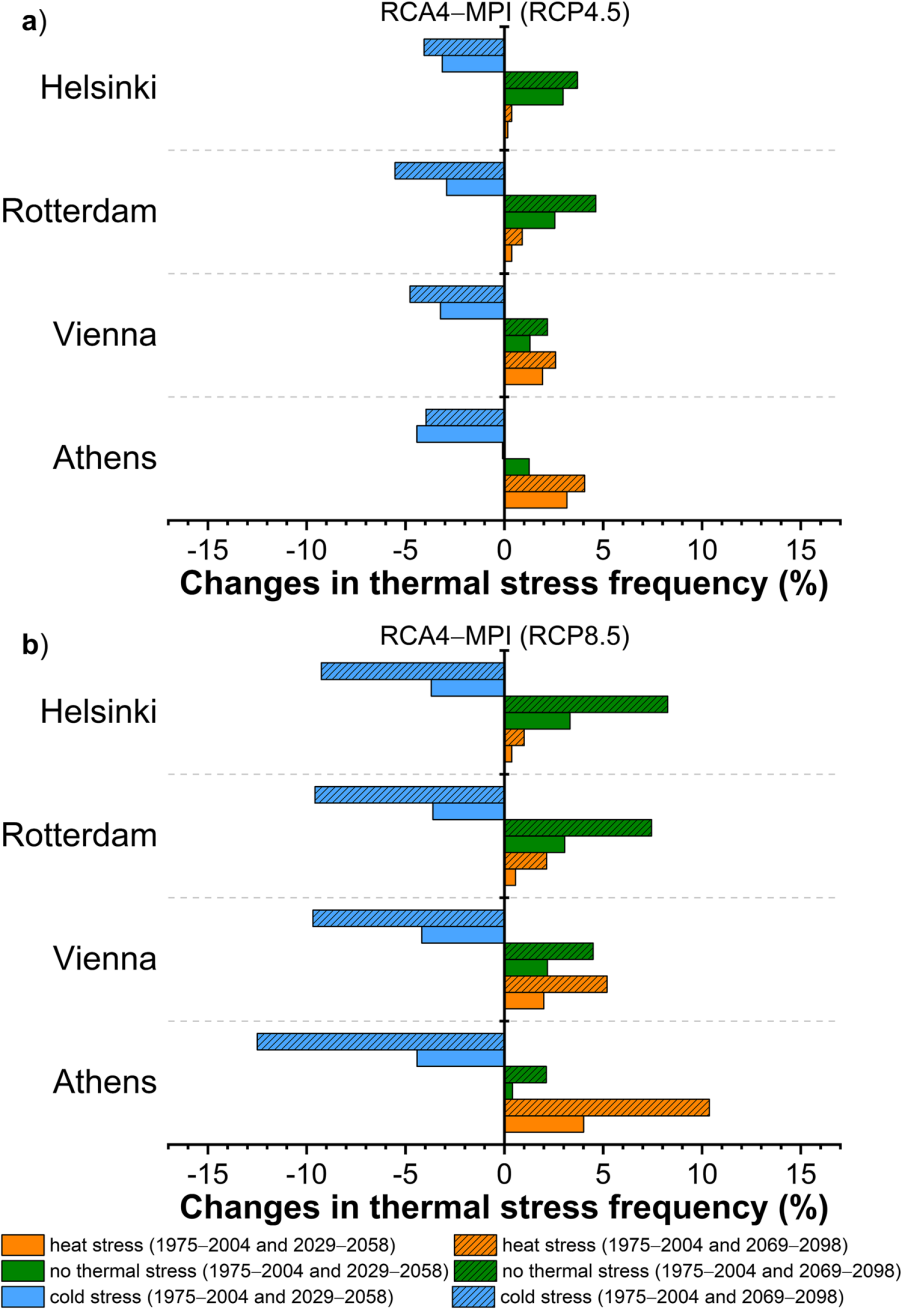
Changes in thermal stress frequency based on UTCI between the reference period (1975 − 2004) and the near future (2029 − 2058) or the distant future (2069 − 2098) according to the RCA4-MPI model for **a**) RCP4.5 and **b**) RCP8.5 in the cities of Helsinki, Rotterdam, Vienna, and Athens

The above results are further highlighted in Figs. 6 and 7 which depict the changes (absolute changes, %) in the frequency of thermal stress categories between the reference period (1975 − 2004) and the two future periods (2029 − 2058 and 2069 − 2098), under both scenarios (RCP4.5, RCP8.5) and according to the RCA4-MOHC and the RCA4-MPI, respectively. Positive changes indicate an increase in the frequency of thermal stress conditions in the future compared to the reference period, while negative changes suggest a decrease. A reduction in the frequency of cold stress conditions is expected in the future climate, consistent across all cities. However, the magnitude of reduction varies strongly, depending on the adopted scenario, the future period and the selected model. According to the RCA4-MOHC model, Helsinki and Rotterdam are expected to experience the highest reduction in the frequency of cold stress in the future (Fig. 6). In Helsinki, the reduction ranges between − 6.2% for the RCP4.5 scenario in the near future and − 15.7% for the RCP8.5 in the distant future. Similar changes are foreseen in Rotterdam, ranging from − 6.7% for the RCP4.5 scenario in the near future to − 16.2% for the RCP8.5 in the distant future. The reduction in the frequency of cold stress conditions in Athens and Vienna is foreseen to be lower by about 0.6 to 3.7% compared to Helsinki and Rotterdam, depending on the scenario and the future period. Simulations based on RCA4-MPI (Fig. 7) show a more modest decrease in the frequency of cold stress compared to RCA4-MOHC, along with some additional differentiations across cities, mainly regarding the cities with the highest reduction.

Heat stress conditions are projected to increase in all cities in the future, according to any model and scenario (Figs. 6, 7), but with distinct differentiations across cities. The greatest increment is expected in Athens (up to + 14.6%, RCA4-MOHC), followed by that in Vienna (up to + 7.6%, RCA4-MOHC) while Helsinki shows the smallest increase (up to + 3.1%, RCA4-MOHC). Although the increase of heat stress conditions according to the RCA4-MPI (Fig. 7) is smaller by about 0.3 to 4.2% in all cities (depending on the adopted scenario and the future period) compared to the projected increase of RCA4-MOHC, the increment pattern between the cities is similar for both models.

As expected, the projected changes in the occurrence of heat or cold-related stress determine the frequency of no thermal stress conditions in the future. According to Figs. 6 and 7, the frequency of no thermal stress conditions shows an increase in the future compared to the reference period in all cities, with the exception of Athens in distant future, where a marginal decrease is projected. The cities expected to experience more frequent no thermal stress conditions in the future are those with lower projected increase in heat stress and vice versa. The greatest increase in the frequency of no thermal stress conditions is expected in Helsinki (up to + 12.7%, RCA4-MOHC), followed by that in Rotterdam (up to + 11.7%, RCA4-MOHC) and Vienna (up to + 4.9%, RCA4-MOHC). On the other hand, Athens shows the smallest changes which is either positive suggesting an increase or negative indicating a decrease of these conditions in the future depending on the scenario, the period and the model.

### Seasonal changes in future thermal stress

**Fig. 8:**
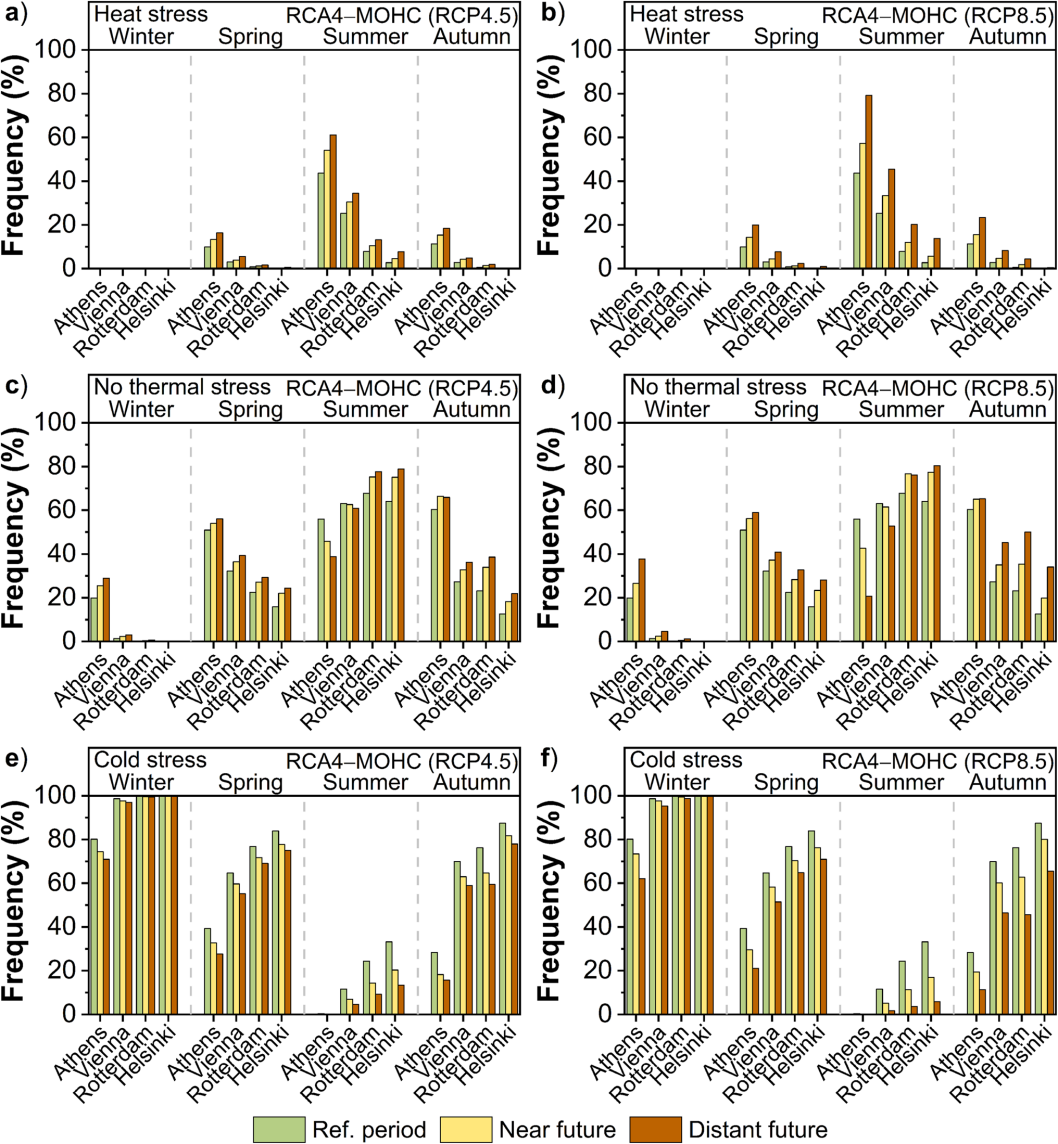
Seasonal frequency of “heat stress” (upper), “no thermal stress” (middle), and “cold stress” (lower) based on UTCI for the reference period (1975 − 2004), the near future (2029 − 2058) and the distant future (2069 − 2098) according to the RCA4-MOHC model for a), c), e) RCP4.5 and b), d), f) RCP8.5 in the cities of Helsinki, Rotterdam, Vienna, and Athens

**Fig. 9:**
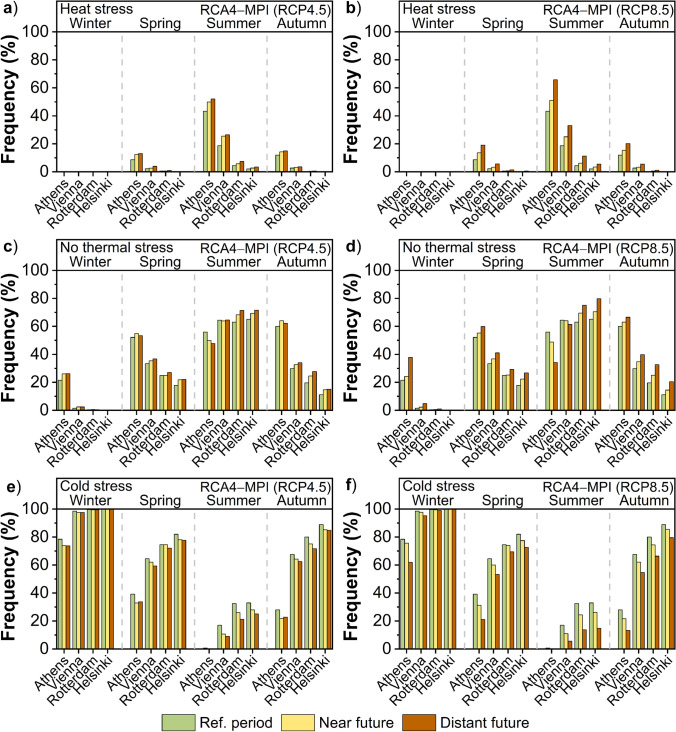
Seasonal frequency of “heat stress” (upper), “no thermal stress” (middle), and “cold stress” (lower) based on UTCI for the reference period (1975 − 2004), the near future (2029 − 2058), and the distant future (2069 − 2098) according to the RCA4-MPI model for **a**), **c**), **e**) RCP4.5 and **b**), **d**), **f**) RCP8.5 in the cities of Helsinki, Rotterdam, Vienna, and Athens

In this paragraph, the seasonal changes in the frequency of heat stress, no thermal stress, and cold stress in the future climate under RCP4.5 and RCP8.5 scenarios are analyzed for the models RCA4-MOHC (Fig. 8) and RCA4-MPI (Fig. 9). Four 3-month seasons are considered, namely, the winter (December to February), the spring (March to May), the summer (June to August), and the autumn (September to November). The frequency of thermal conditions that impose heat stress on the human body is projected to increase mostly during summer in all cities, followed by that of spring and autumn (Fig. 8a, b, Fig. 9a, b). The highest increase in summer is foreseen in Athens, followed by that in Vienna, Rotterdam, and Helsinki. The expected increase in the transitional seasons is more prominent in Athens and Vienna, in contrast to the marginal increase in Rotterdam and the absence of any increase in Helsinki. No changes in the frequency of heat stress conditions were detected in any of the cities, model, future period or scenario, in winter.

As for the conditions of no thermal stress, a considerable increase in the frequency is foreseen in all cities in spring and autumn in the future climate (Fig. 8c, d, Fig. 9c, d). However, the expected increase seems to be more prominent in autumn compared to spring especially for the cities of Helsinki, Rotterdam, and Vienna. The above outcome is further evident in the distant future compared to the near future and for the RCP8.5 compared to the RCP4.5. In winter, the frequency of no thermal stress conditions is expected to increase in Athens, while a slight increase is foreseen in Vienna. No significant changes in the frequency of these conditions were detected in the northern cities of Helsinki and Rotterdam. In summer, the results are significantly differentiated among the cities, revealing an opposite response to the future climate, with increased frequency of the no thermal stress conditions in Helsinki and Rotterdam and decreased frequency in Athens and Vienna.

The frequency of thermal conditions that impose cold stress on the human body is foreseen to decrease in all cities in the transitional seasons of the future climate, though this reduction seems to be slightly greater in autumn than in spring, especially under the RCP8.5 scenario (Fig. 8e, f, Fig. 9e, f). In summer, the highest decrease in the frequency of these conditions is foreseen for Helsinki and Rotterdam, followed by that in Vienna. Since the frequency of cold stress is extremely low during the summer in the present climate of Athens, any changes in the future climate are marginal. On the other hand, the most prominent decrease in the frequency of cold stress during the winter is expected in Athens. A slight decrease is also foreseen in Vienna, while no change is projected for the northern cities of Rotterdam and Helsinki.

## Discussion and conclusions

Even though cities cover only a small proportion (~ 2%) of the Earth’s surface, the population residing in urban areas represents more than half of global population, and it is constantly increasing (United Nations 2019). The urban population in European cities, specifically, has increased by 23.2% in the last seven decades, from 51.7% in 1950 to 74.9% in 2020, and it is expected to further increase reaching 77.5% in 2030 and 83.7% in 2050 (United Nations 2019). Changes in the thermal environment due to global climate change (Coffel et al. 2017; Li et al. 2018), further enhanced by the urban heat island (Founda and Santamouris 2017; Katavoutas and Founda 2019a; Katavoutas et al. 2015; Sarangi et al. 2021; Tan et al. 2010; Van Hove et al. 2015), are expected to pose serious challenges to urban population in the future, in terms of human thermal comfort, health and welfare of people (Founda 2021; Katavoutas and Founda 2019b; Kjellstrom et al. 2016; Matthews et al. 2017; Sheridan and Allen 2015).

The present study has conducted a comparative analysis of observed and projected thermal stress (heat/cold/no thermal stress) across four European cities (Helsinki, Rotterdam, Vienna and Athens), which are representative of different geographical and climatic regions of the continent. The distribution of thermal stress in the current climate varies greatly between the cities, reflecting their climatic and urban heterogeneity. Athens could be considered as the most privileged city during the present climate (reference period), since no thermal stress conditions prevail on average during almost half of the year. The frequency of no thermal stress conditions has been found to decrease with increasing latitude. Cold stress conditions dominate in the cities of Helsinki, Rotterdam, and Vienna throughout the year, while Athens experiences the largest proportion of heat stress. All cities, without exception, are expected to experience a reduction in the annual frequency of cold stress in the future, depending on the model and the emission scenario. While in northern cities (Helsinki, Rotterdam), the reduction in cold stress frequency dominates over the increase in heat stress, thus inducing more neutral (no thermal stress) conditions annually, in warmer cities (like Athens) cold stress reduction is counteracted by the increase in heat stress, thus resulting in negligible changes of no thermal stress conditions in the future. Overall, the projected increase in the frequency of no thermal stress increases with increasing latitude.

The findings of this comparative study come to complement other relevant studies conducted in other cities or regions in Europe that investigate possible future changes in thermal stress levels, such as in German cities (Brecht et al. 2020; Ketterer and Matzarakis 2014; Matzarakis and Endler 2010), in Warsaw (Poland) (Blazejczyk et al. 2013), in Luxembourg (Lokys et al. 2015; Matzarakis et al. 2013), in Gothenburg (Sweden) (Thorsson et al. 2011), in the Netherlands (Molenaar et al. 2016), and in Athens (Greece) (Giannakopoulos et al. 2011; Keramitsoglou et al. 2017; Matzarakis et al. 2014). In the future climate of Warsaw, the study of Blazejczyk et al. (2013) showed an increase of 9% in the number of days with at least strong heat stress in summer and a decrease up to 30% in the number of days with at least strong cold stress in winter. According to the study of Molenaar et al. (2016), heat stress hours are expected to more than double in 2050 in several cities in the Netherlands. The findings of Thorsson et al. (2011) in Gothenburg indicate higher decrease in the number of hours with strong/extreme cold stress compared to the increase in the number of hours with strong/extreme heat stress in the future. The latter outcome is in accordance with our results in the Scandinavian city of Helsinki, and our study further highlights how this pattern that is expected in northern cities is changing when moving to lower latitudes and warmer cities. Previous studies in the city of Athens have shown that the number of consecutive days with high thermal discomfort is expected to increase both in the near future (by about 12 days) and in the distant future (by 32 days) (Giannakopoulos et al. 2011; Keramitsoglou et al. 2017).

Our findings also suggest that although future projections are expected to partly bridge the gap between the less-privileged cities (with respect to annual frequency of optimal thermal conditions) like Helsinki and Rotterdam and the more privileged ones like Athens, the former will still lag behind on an annual basis. On a seasonal basis, the future projections indicate a considerable increase in the exposure time under ideal conditions in the shoulder seasons of spring and autumn in all cities, more prominent in autumn especially for Helsinki and Rotterdam. In winter, the frequency of no thermal stress conditions is expected to increase in cities like Athens and to a much lesser extent in Vienna, but no change is foreseen for cities like Helsinki and Rotterdam in the future. A different pattern is expected in summer between the cities, with less frequent ideal conditions in Athens and Vienna and more frequent in Rotterdam and Helsinki.

The seasonal analysis of thermal stress across the selected European cities provides evidence on the deterioration or improvement of thermal conditions, which are not uniform among the cities and thus, more targeted actions should be taken into account when adaptation strategies for key sectors like health or tourism are planned for each city. The deterioration of thermal conditions during summer especially in the Mediterranean city of Athens or even in Vienna could lead to the adoption of advanced mitigation technologies to attenuate the heat stress of their residents (Dandou et al. 2021; Santamouris and Yun 2020). In addition, the improvement of thermal conditions in the transitional seasons in Athens could lead to a shift of future tourist flows for city breaks from the summer months to spring and autumn. Possible redistribution in tourist flows due to climate change has also been reported especially in popular tourist and beach destinations around the world (Amelung and Nicholls 2014; Amengual et al. 2014; Demiroglu et al. 2020; Grillakis et al. 2016; Katavoutas et al. 2021; Nastos and Matzarakis 2019). The northern European cities like Helsinki and Rotterdam might be more attractive in the future during the summer months due to the improvement of thermal conditions, resulting in new opportunities for the tourism industry. At the same time, the urban population of these cities is expected to experience more favorable conditions in terms of thermal comfort.

Cities are considered as the main contributors in greenhouse gas emissions and they also consume about three-quarters of primary energy globally (Creutzig et al. 2015; Seto et al. 2014). Yet, emissions from fuel combustion and electricity in buildings have been found to contribute between 60 and 80% of total emissions in North American and European megacities (Wei et al. 2021). In addition to rapid urbanization and economic growth, potential future changes in the thermal environment of cities will probably pose further challenges in the energy sector, affecting the energy demands for cooling or heating purposes.

It is noteworthy that adaptation, acclimatization, expectations, and behavior are all essential factors in the perception of the thermal environment (Nikolopoulou and Steemers 2003). Therefore, the effect of the same thermal conditions on urban populations in a colder temperate climate could possibly be more severe than on populations of cities like Athens, where people are more accustomed to excessive heat and at the same time, most of the buildings are already equipped with air conditioners. In this study, the original scale of UTCI (Bröde et al. 2012) was adopted, assigning the different cold and heat stress categories. However, it is understood that northern or southern Europeans have likely different levels of acclimatization in cold or heat stress. For instance, the category corresponding to slight cold stress could very likely represent neutral conditions (no thermal stress) for cold cities, like Helsinki. As an example, if this category is assigned as neutral (no thermal stress) for cold cities, the percentage of cold stress in winter in the near future would change from 99.9 to 98.8% in Helsinki, whereas the conditions of no thermal stress in winter would change from 0.1 to 1.2% (MOHC, RCP4.5) (Fig. 8c, e). Although this would quantitatively differentiate some of the results, the qualitative pattern of changes on thermal stress in the future climate would still exist.

In our analysis, the implications of climate change on thermal conditions (cold stress, heat stress, no thermal stress) were inspected in four European cities with different background climates, focusing on the better understanding of projected future changes. This could be used as a dynamic policy tool providing evidence on the deterioration or improvement of thermal conditions, which are not uniform among the cities and thus, aid the planning of appropriate health adaptation strategies to reduce thermal related morbidity and mortality in each city.
